# Synthesis of Ni/Al_2_O3 catalysts via alkaline polyol method and hydrazine reduction method for the partial oxidation of methane

**DOI:** 10.3906/kim-2012-46

**Published:** 2021-08-27

**Authors:** Ezgi BAYRAKDAR ATEŞ, Tuba GÜRKAYNAK ALTINÇEKİÇ, Mehmet Ali Faruk ÖKSÜZÖMER

**Affiliations:** 1 Energy Systems Engineering Department, Faculty of Engineering, Yalova University, Yalova Turkey; 2 Department of Chemical Engineering, İstanbul University-Cerrahpaşa, İstanbul Turkey

**Keywords:** Alkaline polyol method, hydrazine reduction, nickel, nanoparticles, catalytic partial oxidation of methane

## Abstract

Nickel catalysts supported on γ-Al_2_O_3_ were synthesized in the presence of polyvinylpyrrolidone (PVP) using both alkaline polyol method and hydrazine reduction method while fixing the weight ratio of [(PVP)]/[Ni(CH_3_COO)_2_·4H_2_O] at 2. The effects of hydrazine [N_2_H_5_OH]/[Ni] and [NaOH]/[Ni] molar ratios on the structural properties of the catalysts were characterized by transmission electron microscopy (HRTEM) and by X-ray diffraction (XRD). The average of monodispersed Ni nanoparticles ranged between 8.0 and 13.0 nm. The catalytic tests were performed for the partial oxidation of methane in the temperature range of 600–800 °C under a flow rate of 157,500 L kg^–1^ hr^–1^ with CH_4_/O_2_= 2. At the molar ratio of [NaOH]/[Ni] = 2, the resultant nickel nanoparticles on alumina was established completely without impurities; thus, it demonstrated the highest catalytic activity, 88% for CH_4_ conversion, and H_2_ selectivity, 90.60%. The optimum [N_2_H_5_OH]/[Ni] ratio was determined as 4.1, which means a good catalytic performance and 89.35% selectivity to H_2_ for the partial oxidation of methane.

## 1. Introduction

Nanosized nickel particles have been mainly preferred as catalysts in the catalytic partial oxidation of methane (CPOM) which is one of the basic H_2_ production processes. Although noble metals are very stable and active, low availability and high cost make the use of noble metals difficult. Both noble and non-noble metal-based catalysts have long been investigated in detail for the methane partial oxidation process [15]. -Although several noble metals have been used as catalysts for CPOM, most studies have focused on Rh, Ru, and Pt [6] among which Rh stands out as the most active for the CPOM process. Noble metal catalysts exhibit higher activity and stability. However, they have characteristics such as high cost and less readily available than their non-noble-metal counterparts [7]. When it comes to non-noble metal based catalysts the most preferred catalysts for the CPOM, these mainly based on transition metals, mainly supported Ni and Co, have advantages to noble metals-based catalysts thanks to their superior performance, abundance and relatively low cost [810]. Cobaltbased catalysts appear as good alternatives due to their .- more resistance against deactivation by coking [11,12]. However, Co catalysts has lower activity [11]. and also could be oxidize easier than when using Ni [13]. Moral et al. [14] studied over a series of Co/Mg-Al catalysts and proposed different approaches to improve the catalyst stability of Co catalysts for CPOM.

Different proportions of metal loading on the support strongly affect the catalyst properties such as surface area, crystal structure, metal support interaction, and metal particle size and hence affect the performance of the catalyst. Several research groups have widely investigated Ni based catalysts and found that supported Ni catalysts are highly active and selective for this reaction [3,15]. These catalysts can be prepared with a high metal loading (1030% by weight) because of their cheapness. Khajenoori et al. [16] studied the effect of Ni-CeO-_2_/MgO catalyst with 5–15% by weight Ni loading. They found that CO_2_ and CH_4_ conversions showed significant increases when Ni loading increased up to 10% by weight, but catalyst performance decreased with Ni loading of more than 10% by weight due to the formation of large Ni crystals with low Ni dispersion. However, the metal dispersion becomes poor as the metal content increases due to the metal particle agglomeration on the support surface. Most of the literature studies are aimed at understanding the reaction mechanism and minimizing catalyst deactivation processes such as transformation of nickel (forming inactive phases, e.g. NiAl_2_O_4_) and coke deposition (as filaments or layered structures). Most of the studies showed that 10% Ni/Al_2_O_3_ catalysts exhibited always higher than 95% conversion of CH_4_ with 100% H_2_ selectivity at high WHSV values by using O_2_ [17]. 

The effect of the support material was also investigated on the catalytic performance for CPOM reaction [18,19]. The use of rare earths as a support material (TiO_2_, Fe_2_O_3_, Al_2_O_3_, SiO_2_, Cr_2_O_3_, ZrO_2_, La_2_O_3_, MnO, etc.) contributes to the oxygen storage /release capacity by oxidizing the accumulated carbon and causes a reduction in sintering by creating an effective interaction with the active phase [7,2022] emphasized that especially heat-resistant metal oxides were chosen as the most support material- and they also demonstrated how the support basicity greatly influenced both the H_2_/CO ratio and the carbon deposition. By using metal oxides, various performances of catalysts such as mechanical strength, microporous structure, reactivity and stability can be significantly improved [12,23]. Supports such as ZrO_2_, MgAl_2_O_4_ and Al_2_O_3_ are frequently used in industry for many catalytic reactions and have desirable properties such as high surface area and high mechanical strength. We used γ-Al_2_O_3_, which is one of the typical supports for Ni catalysts, with the properties mentioned in this study.

As we mentioned aboveSo as nickel is a potential substitute of noble metals as active phase, but it shows deactivation due to the coke formation, sintering or phase transformation during its use in high-temperature CPOM reaction [6]. Literature studies show that the presence of well-dispersed small metallic particles (< 10 nm) on support and its modification with basic oxides is necessary to prevent coking and sintering problems [24,25]. The chemical mechanism during nanomaterial synthesis can avoid three significant problems diffusion, impurities and agglomeration [26]. In the literature many methods such as hydrothermal [28], chemical control reduction [29], electrochemical [30], ethanol-water system (EWS) [31], sol-gel [32] and polyol [3335] , such as have been developed to synthesize Ni nanoparticles [27]-.

As a nanomaterial preparation method, the solution reduction method such as polyol method could exhibit advantages compared to other synthesis methods. The polyol process is one type of solution reduction method defined as an alcohol reduction method. It is a cost-effective, feasible and environmentally friendly method [36,37] . This process could be used to synthesize monodispersed cobalt, nickel, copper and precious metals in the micrometer and submicrometer range [38,39] without agglomeration. Polyol method is also appropriate for scaling up compared to any other processes that consist of the high cost and noxious compound consumption as well as more complicated procedure [40]. In conventional polyol process, liquid polyols such as ethylene glycol, 1,2-propanediol, diethylene glycol, or triethylene glycol act as both solvent and mild reducing agent. In a typical procedure, the metal precursor is suspended or dissolved in polyols and the resultant solution is heated up under reflux, and then the metallic particles are formed by redox reaction. During metal nanoparticle synthesis, polymer stabilizers such as polyvininylpyrrolidone (PVP) can be used to adjust the size, shape and morphology as well as prevent aggregation [41]. The optimum synthesis parameters such as the reaction time, reduction temperature, reducing agent type, ratio of reducing agent to surfactant, and pH could be adjusted quickly to keep the nanoparticles’ size and shape under control of [38]. Researchers indicate that the reduction rate, crystallite, and particle sizes of the synthesized materials are mainly affected by the synthesis parameters [42]. Among the possibilities mentioned in the literature most of the studies focused on the variations of the particle characteristics by changing the source material’s concentration [43], hydrazine [44], and the surfactant [29]. In the absence of PVP or any protective agent (surfactant), products mostly form in micrometer size [37].Therefore, PVP was used as a stabilizer in this study. The basic environment required for the Ni(II) reduction mechanism of the polyol process was provided by the addition of NaOH. Basicity is one of the important parameters that has a pronounced effect on the structure, shape and purity of Co and Ni particles [45]. NaOH accelerates the metal hydroxide formation and dehydration of glycol to aldehyde [36]. The reduction step can be assisted by adding an additional reducing agent such as N_2_H_5_OH (hydrazine hydroxide) along with NaOH. In this way, the formation of smaller metallic particles is usually achieved in the presence of a capping agent such as PVP. Synthesis carried out in this way is called the modified polyol process. For the hydrazine reduction method, the reduction is achieved at low temperatures (nearly 80C) and takes a shorter time compared to the conventional polyol method.

Therefore; in this study we used the advantages of two types of polyol processes and reported on the effects of reaction parameters on Ni nanoparticles’ morphologies and tested their performances in the catalytic partial oxidation of CH˚_4_ to syngas in the medium-high temperature range 600800C at atmospheric pressure. The influence of NaOH and N-˚_2_H_5_OH on the physical properties of the supported Ni catalysts were investigated by using X-ray diffraction (XRD), high-resolution transmission electron microscopy (HRTEM), atomic absorption spectroscopy (AAS) and thermal gravimetric analysis (TGA).

## 2. Materials and methods

γ-Al_2_O_3_ (BET surface area =132 m^2^/g, average pore size of approximately 23.9 Å, purity above 98%) was obtained from Alfa-Aesar and used as the catalyst support throughout this study. Nickel acetate (Ni(OAc)_2_.4H_2_O (Aldrich ≥ 98%), ethylene glycol (CH_2_OHCH_2_OH) (EG) (Sigma-Aldrich ≥ 99.5%), Polividon 25, (C_6_H_9_NO)_n_ (PVP) (Merck) with molecular weight (Mw), 10,000 g/mol, sodium hydroxide (NaOH) (Merck), hydrazine hydroxide (N_2_H_5_OH) (Merck wt.80%) and acetone were used without further purification.

### 2.1. Synthesis of supported Ni catalysts using alkaline polyol method and hydrazine reduction method

**Figure 1:**
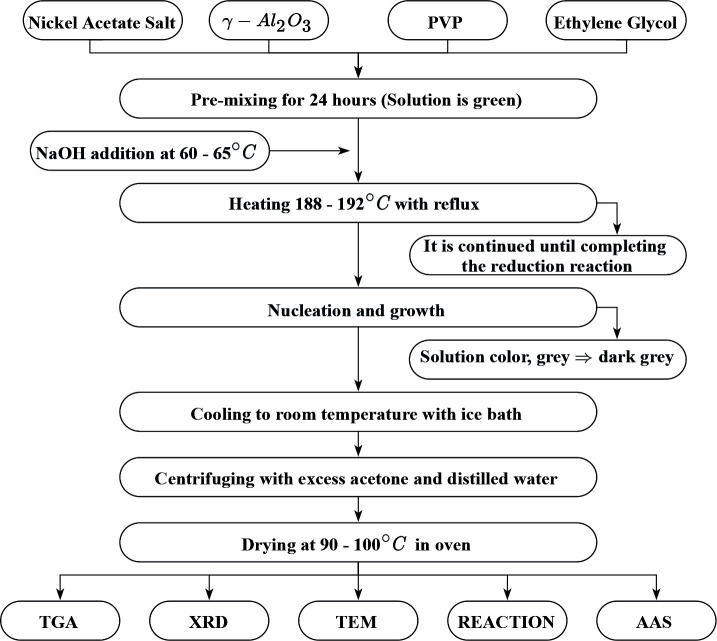
Flow chart of alkaline polyol method.

The procedure of the alkaline polyol method is shown in Figure 1. A required amount of the Al_2_O_3_ was put into an EG-PVP-metal precursor solution (Ni) and stirred for 24 h using a magnetic stirrer in a three-neck round-bottomed flask with reflux at 30°C. The nickel acetate salt concentrations were fixed at 0.05 Mours and calculated to provide the 10 wt.% Ni loading for each sample. According to our recent studies, the PVP amount was fixed at the optimum ratio of PVP/Ni (w/w) 2:1 [41]. In the alkaline polyol method, after the pre-mixing period, the suspension was then heated to 6065°C, and NaOH added at this temperature. When the reaction temperature reached the EG’s boiling point, the reaction was maintained until the dark gray suspension formed. This dark gray homogeneous colloidal solution was then quickly cooled down to room temperature in ice bath. The synthesized catalysts were separated by centrifuging, then washed by using excess acetone and distilled water to remove organic or other impurities, and then dried at 90–100°C in a furnace for 1518 h. The catalysts prepared with varying NaOH/Ni ratio were denoted as Ni/γ-Al --ours_2_O_3_-aNaOH where “a” refers to the ratio of NaOH/Ni (mole/mole). 

**Figure 2:**
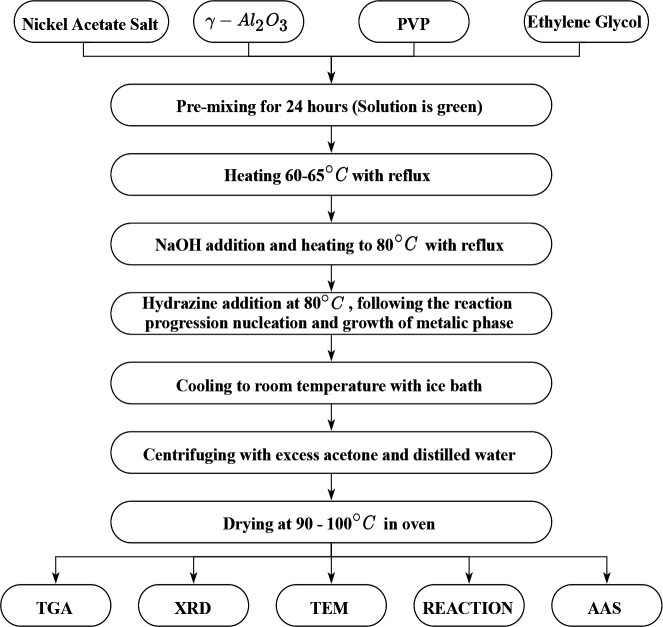
Flow chart of hydrazine reduction method.

A similar procedure for the hydrazine reduction method flowchart is shown in Figure 2. N_2_H_5_·OH of 80% concentration was added into the slurry and agitated at the desired temperature at 7080°C to reduce the Ni(II) ions to metallic Ni on the γ-Al-;_2_O_3 _support. The progress of the reaction was followed by the transformation of the solution color from green at first to gray then dark gray. Then, the synthesized catalysts were separated and dried as in the alkaline polyol method. The catalysts prepared with different N_2_H_5_OH/Ni ratio were denoted as Ni/γ-Al_2_O_3_-bN_2_H_5_OH where “b” refers to the ratio of N_2_H_5_OH/Ni (mole/mole). The most appropriate N_2_H_5_OH/Ni and NaOH/Ni molar ratio values were determined with preliminary studies and investigating literature. When the literature studies are examined, the pH value of the system has an effect on the morphology of the nanoparticles such as Ni, Co and Cu [46].

In cases where the NaOH concentration is below 0.25 M, undesired anisotropic growth can occur [47]. The NaOH concentration was adjusted so that it did not fall below 0.25 M. 

The chemical reaction that is occurred in the hydrazine reduction method can be formulated as below in Eq. (1) and Eq. (2) [48]:

(1)Ni(Ac)2+2NaOH→Ni(OH)2+2NaAc(Formaton of Nickel Hydroxide)

(2)Ni(OH)2+1/2N2H4→Ni+1/2N2+2H2O(Reduction Step)

Alkalinity is required for Ni(II) reduction, due to its low oxidation potential and, therefore, the ‘‘pH’’ value for the formation of the particles is very important. It helps to form Ni(OH)_2_ precipitates from Ni(Ac)_2_ in EG and provides the alkalinity necessary for the use of N_2_H_5._OH as a reducing agent [42]. 

### 2.2. Catalyst characterization

The catalysts were characterized by using atomic absorption spectrophotometer (AAS), high-resolution transmission electron microscope (HRTEM), X-ray diffraction (XRD), and thermal gravimetric analysis (TGA). Actual ickel loadings of the as- synthesized catalysts on alumina support were determined with Analyst 200 ﬂame (Perkin Elmer) AAS equipment. Nickel particle sizes and morphologies of the catalysts were characterized by using JEOL 2100 LaB6 HRTEM. Crystallographic structure and crystallite size of the synthesized Ni nanoparticles were identified using Rigaku D/Max-2200 diffractometer with Cu-Kα (λ = 0.154021 nm) radiation source in the angle range of 2θ= 1090N- .The mean crystallite sizes of metallic Ni were determined with the Scherrer equation, where the “K” constant was considered 0.9 and “b” was the FWHM of the Ni (200) at 51.7°. Ni metal dispersions (D%) on the alumina support were calculated assuming the particles are spherical. Using XRD crystallite sizes of Ni(200), the equation given below was used to define and show fresh catalysts’ metal dispersion in Eq. (3) . 

(3)D(%)=971dNi

Just before the catalytic activity measurements, the catalyst was pre-treated at 450°C for 4h under H_2_/N_2_(1:4) ﬂow in Micro-reactor (CATLAB)-MS(QIC-20) system (Hiden Analytical). Thermogravimetric analysis (TGA) was used under a gas flow rate of 40 ml/min up to 900°C at 5°C/min for whole synthesized catalysts and post-reaction catalysts. Physisorption isotherms were obtained by the adsorption of N_2_ at 77 K in a Quantachrome Nova 3200e. The samples were dried at 100 °C under vacuum for 3 h in order to eliminate the adsorbed species. The pore volume and specific surface area of catalyst samples were calculated by the BarretJoynerHalenda (BJH) and Bru- nauereEmmetteTeller (BET) methods, respectively.

### 2.3. Catalytic activity and selectivity tests

The activity and selectivity measurements of catalysts were performed at atmospheric pressure in a quartz microreactor (0.4 cm inner diameter and 19 cm length) accoutered with a thermocouple in the center of a catalyst bed. That equipped system was connected to the GC (HP 5890) for the measurements and integrated line was heated with a heat tape to avoid the condensation of water in the line. Before the effluent stream entered the GC, it was cooled in a condenser in order to remove the formed water. After this step, the reactant gas mixture (CH_4_/O_2_/N_2_ =30/15/60) was transferred into the reactor with the ﬂow rate of 105 mL/min (GHSV=157,500 L kg^−1^ h^−1^). Efﬂuents were analyzed from 600°C to 800°C with 100°C temperature intervals. During the activity tests, carbon balances were always between 95-100%. For the long term catalyst stability of the CPOM catalysts prepared with the modified polyol method, the more practical “accelerated” thermal stability test was applied instead of the long (> 50 hours) time-on-stream tests. More than the stoichiometric ratio of methane was used to determine the carbon resistance. Stability tests were performed at 800 °C for 10 hours using ratios of CH_4_/O_2_/N_2_ = 47/12/47.

Activity and selectivity results were calculated by using the equations given below Eq. (4), Eq. (5), and Eq. (6) [49]:

(4)CH4conversion:XCH4=FCO(out)+FCO2out)FCO(put)+FCO2(out)+FCH4(out)x100

(5)H2selectivity:SH2=FH2(out)2x(FCO(out)+FCO2(out))x100

(6)CO selectivity:SCO=FCO(out)FCO(out)+FCO2(out)x100

## 3. Results and discussion

### 3.1. Catalyst characterization

Ni particles were prepared by reduction of Ni(II) acetates in a solution of NaOH and hydrazine in EG. Ni^0^ phase as active metallic component plays a crucial role in determining the activity and stability of a catalytic system. The Atomic Absorption Spectroscopy (AAS) analysis results of the Ni/γ-Al_2_O_3_ catalysts synthesized with alkaline polyol process at different NaOH/Ni molar ratios are given in Table 1. 

**Table 1:** Atomic absorption spectroscopy (AAS) results for Ni loadings, crystallite sizes, particle sizes and Ni dispersion.

Catalyst	Reaction temperature (°C)	Ni (w%)detected	Loading efficiency (%)	Crystallite size (nm)a	Particle size (nm)b	Ni dispersion(%)c
None	196	7.9	79.0	9.04	8.0	10.74
1NaOH	192	-	-	15.2	-	6.40
2NaOH	190	6.0	60.0	13.5	8.5	7.20
4NaOH	188	5.0	50.0	13.5	-	7.20
2PVP-2NaOH						
4.1N2H5OH	90	6.8	68.0	8.03	8.4	12.13
8.2N2H5OH	88	7.4	74.0	7.20	-	13.50

Catalyst: Ni/γ-Al2O3-2PVPa calculated by Scherrer Equation d= b calculated from TEM resultsc XRD crystallite sizes of Ni(200)

It could be found that the actual Ni loading amount onto alumina support is below the nominal value for all synthesized catalysts. The actual Ni loading amounts onto alumina support were 6.0, 5.0 wt.% for alkaline method and 6.8, 7.4 wt.% for hydrazine reduction method, respectively. Metal particles can diffuse into the pores of the catalyst support and can grow in size, and so metal loading levels decreased due to partial blockage of the pores [41, 50, 51]. Our results were compatible with these approaches, despite no linear correlation with the changing NaOH amount in Table 1. Metal Ni loading ratios obtained as a result of the reactions carried out in the presence of hydrazine are 24% better than catalysts using only NaOH. Among the prepared catalysts, the lowest Ni amount was 5.0 (wt.% ) for Ni / γ-Al_2_O_3_-2NaOH, while the highest Ni loading was 7.4 (wt.% ) for Ni / γ-Al_2_O_3_-2NaOH-8.2N_2_H_5_OH catalyst. In both different methods, Ni particles were loaded on the support. Based on the surface area of the support material used, the pore diameter and of course the pore blockage caused by the PVP used as a surface active agent, Ni loading was obtained below the nominal values. The difference between the actual and theoretical loading values in the AAS analysis is thought to be due to the lowering of the metal support interaction due to the added NaOH and PVP, as well as the reduction of some of the Ni salt remaining in the solution. In addition, during the hydrazine reduction, the reaction medium is mixed with a small magnet by applying a magnetic field from the outside in order to be completely homogeneous. It is thought that the magnetic interaction formed by the metal Ni particles showing good magnetic properties affects the deposition of Ni nanoparticles on the support material. Nik Roselina and Azizan [27] explained that the increase in hydrazine ratio may cause more nucleus formation and consequently agglomeration. Wu et al. [52] stated that when working in the range defined as low hydrazine concentration ([N_2_H_5_OH]/[Ni] <12), the reduction rate of the Ni salt is low and therefore the particles grow as the reduction progresses. Although they stated that it would be very difficult to reduce below this concentration value, the reduction process started in 2-3 minutes in the presence of lower hydrazine ratios (such as 4.1 and 8.2) as well as alumina support and PVP.

**Figure 3:**
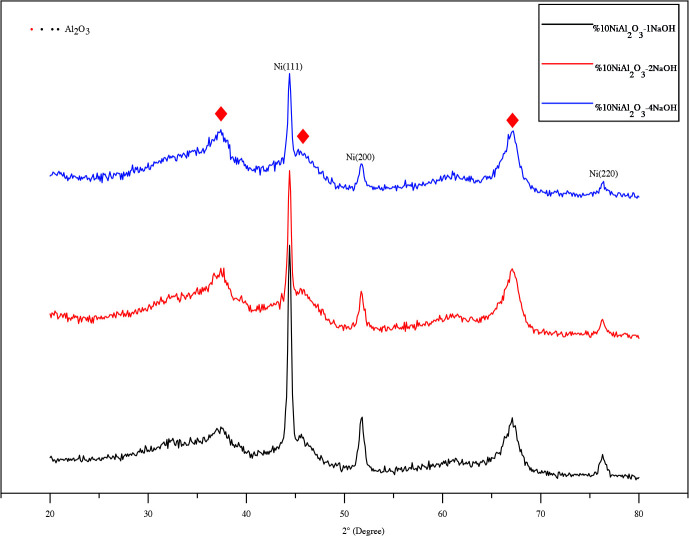
XRD patterns of the Ni/γ-Al_2_O_3_ catalysts with varying NaOH/Ni molar ratios.

**Figure 4:**
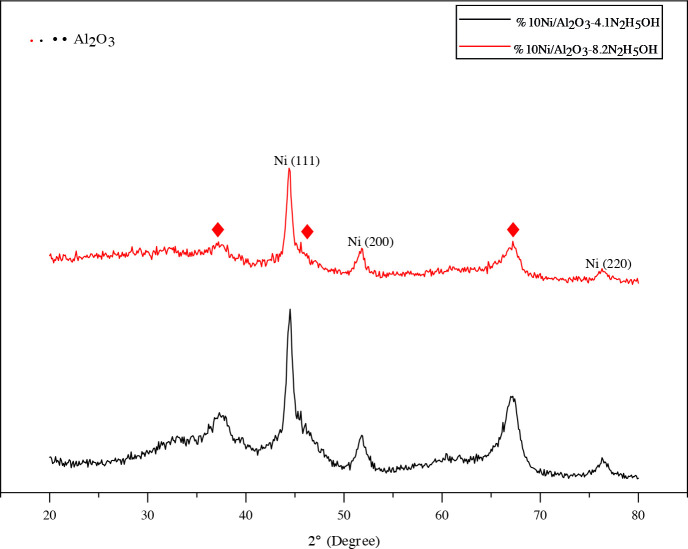
XRD patterns of the Ni/γ-Al_2_O_3_ catalysts with varying N2H5OH/Ni molar ratios.

The XRD patterns of the resultant products synthesized with a varying molar ratio of NaOH/Ni from 1 to 4 by alkaline polyol method are given in Figure 3, and other catalysts prepared with a varying molar ratio of N_2_H_5_OH/Ni 4.1 and 8.2 by hydrazine reduction method are shown in Figure 4. All catalysts showed peaks for metallic Ni (PDF 87-0712), but other Ni-based phases were not found. In basic solutions, a solid intermediate phase precipitates in the medium at high temperature (above 150 °C) before metal ion reduction [38], but at the end of the reaction (with the complete oxidation of the EG) the pure metallic Ni phase precipitated on the support material. Furthermore, the three diffraction peaks at 37.4, 46.1 and 67.2˚ belong to γ-Al_2_O_3_ phase (PDF 04-0880).The Ni peak at 2θ=51.6˚ in Ni/γ-Al_2_O_3_-2NaOH appears more broadly compared to Ni/γ-Al_2_O_3_-1NaOH, thus thereby indicating well dispersion of Ni crystallites [52]. It was observed that the intensity of these peaks decreased due to the increase in the percentage of NaOH ratio and therefore the nickel particle size decreased. In Figure 4, however, the same Ni peak at 2θ=51.6˚ in Ni/γ-Al_2_O_3_-8.2N_2_H_5_OH seems to be more broadly than peaks belonging to products synthesized using NaOH. 

**Figure 5:**
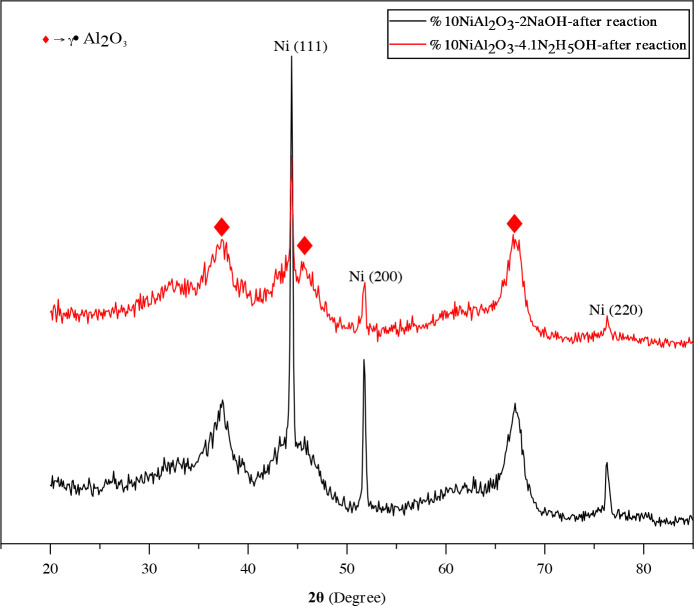
XRD patterns of the spent Ni/γ-Al_2_O_3_-2NaOH and Ni/γ-Al_2_O_3_-4.1N_2_H_5_OH catalysts.

The crystallite sizes of Ni particles, calculated by the Scherrer’ law, are given in Table 1. The average crystallite size was between 7 and 15 nm and the smallest crystallite size appeared in catalyst prepared by hydrazine reduction method. The presented XRD analysis results clearly show the reduction in crystallite sizes of Ni-based catalysts as a function of the increase in the alkalinity of the solution and in the reducing agent content. XRD patterns of spent catalysts in the 2θ range of 10–90º are shown in Figure 5. As shown in Figure 5 broad peaks appeared at 37.29, 44.36, 66.95° corresponding to γ-Al_2_O_3_ (PDF 04-0880). The characteristic peaks for metallic Ni particles are at 2θ = 44.39°, 51.89°, and 76.32° associated with the (111), (200), and (220) reflections for the two spent catalysts. No characteristic peaks (2θ = 37.2°, 43.2° and 62.8°, PDF Card No. 47-1049) corresponding to NiO and no characteristic peaks ( 2θ = 31.4, 44.9, 55.7, 59.6, and 65.5º PDF Card No.10-0339) corresponding to NiAl_2_O_4_ were observed from the XRD pattern of the spent catalysts. This shows that Ni metal salts are successfully reduced to metallic Ni by the modified polyol process and the oxidation degree of nickel does not change during the CPOM reaction. The average crystallite size for both the samples Ni /γ-Al_2_O_3_-2NaOH and Ni/γ-Al_2_O_3_-4.1N_2_H_5_OH was calculated using Scherrer formula and found 8.46 and 8.03 nm respevtively. It was observed that both samples showed very similar crystallite sizes with fresh catalysts after CPOM reaction. Despite the long reaction time, the absence of carbonization in these catalysts after the reaction shows that stable Ni catalysts can be prepared by the modified polyol method.

**Figure 6:**
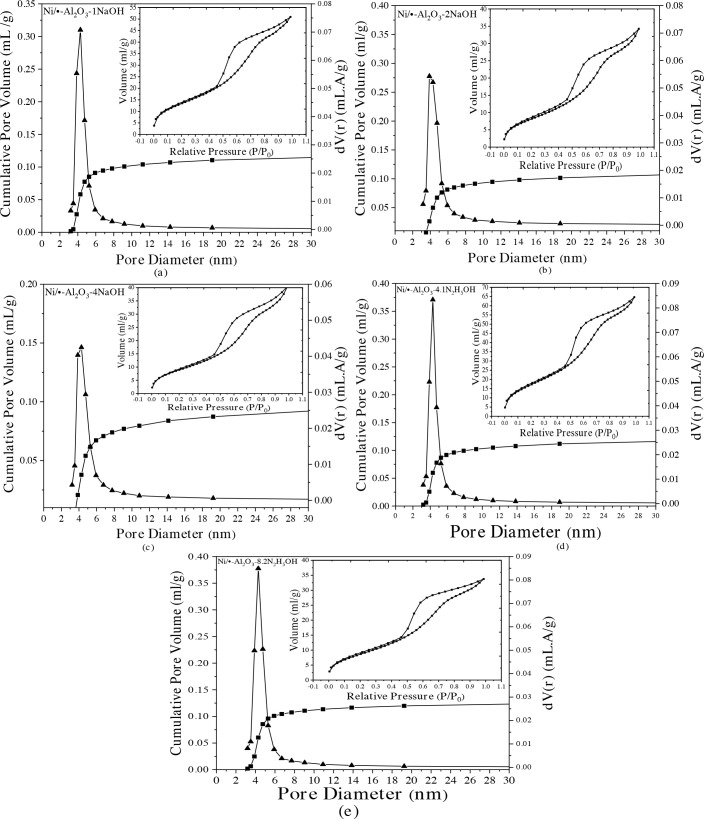
N_2_ adsorption/desorption isotherms and pore-size distributions of the supported Ni catalysts.

N_2_ adsorption-desorption isotherms and pore-size distributions for the catalysts prepared with different N_2_H_5_OH/Ni and NaOH/Ni ratio were given in Figure 6 and the BET (Brunauer-Emmett-Teller) surface area, pore volume and average pore diameter results were given in Table 2. According to the IUPAC (International Union of Pure and Applied Chemistry) classification, the samples represent Type 4 isotherms with the hysteresis loop generally obtained for mesoporous (2–50 nm) structures. The highest BET surface area (79 m^2^/g) and the highest pore volume was obtained when hydrazine is used as reducing agent with NaOH/Ni ratio was 2. It was observed that the surface areas of the Ni/γ-Al_2_O_3_ catalysts with changing NaOH/Ni ratios presents a slight decreasing trend. The BET surface area decreases slowly from 74.8 m^2^/g of Ni /γ-Al_2_O_3_-1NaOH to 51.3 m^2^/g of Ni /γ-Al_2_O_3_-4NaOH. All of the samples were mesoporous, showed unimodal pore-size distribution. The average pore diameter of Ni/γ-Al_2_O_3_ catalysts is in the range of 3.9–4.3 nm (Table 2), while the average Ni crystallite diameter calculated by Scherrer equation from XRD is over 7 nm, indicating that Ni species are dispersed mainly on the γ-Al_2_O_3_ surface without entering into the mesoporous structures of γ-Al_2_O_3_. Therefore, it is possible that partial blockage of Ni particles on the surface of γ-Al_2_O_3_ results in the decrease of BET surface area and pore volume [53].

**Table 2:** The BET surface area (SBET), pore volume (Vp), average pore diameter (Dp) of supported Ni catalysts.

Catalyst Ni/γ-Al2O3-2PVP	SBET (m2/g)BET surface area	Vp (mL/g)Total pore volume	Dp (nm)Average pore diameter
1NaOH	74.84	0.119	4.290
2NaOH	64.29	0.113	3.892
4NaOH	51.24	0.098	4.276
Ni/γ-Al2O3-2PVP-2NaOH			
4.1N2H5OH	78.93	0.121	4.295
8.2N2H5OH	78.94	0.127	4.290

**Figure 7:**
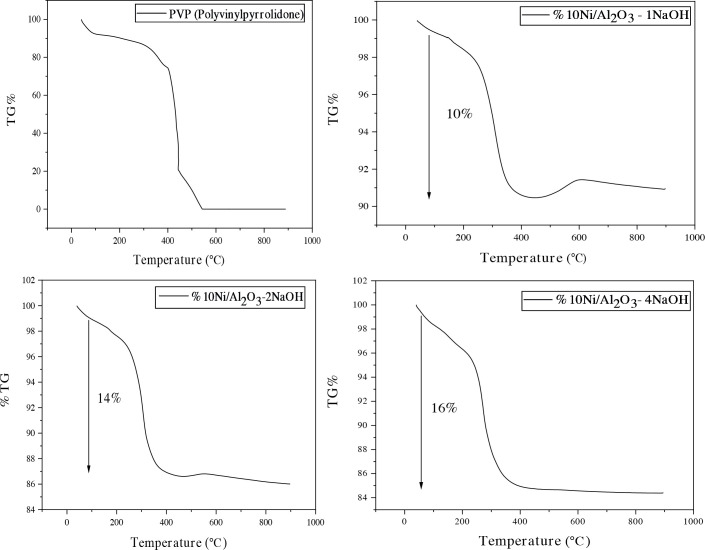
TG analysis of catalysts belong to only PVP and catalysts prepared with varying NaOH/Ni molar ratios.

The TGA curves of catalysts prepared using NaOH and pure PVP are presented in Figure 7. TGA analyzes of the catalysts prepared with the modified polyol method are similar, and their weight loss varies between 10-16% depending on the composition. As the ratio of NaOH content increased, the amount of weight loss increased. The TGA thermogram in Figure 7 shows minor weight loss of 1.94% between 50 and 160 °C due to the loss of physically adsorbed water. The major weight loss of 16% in the temperature range 160–500 °C is due to the combustion of PVP and EG adsorbed in the sample. 

In order to better understand the effect of PVP and NaOH on weight losses occurring in catalysts, thermogravimetric analysis was performed on PVP and non-NaOH catalysts synthesized under the same conditions. As seen in the Figure 7, the lowest weight loss among the catalysts occurred in the Ni/γ-Al_2_O_3_-1NaOH catalyst and, as in the others, approximately 1% weight increase occurred due to the formation of NiO above 560–590 °C.

**Figure 8:**
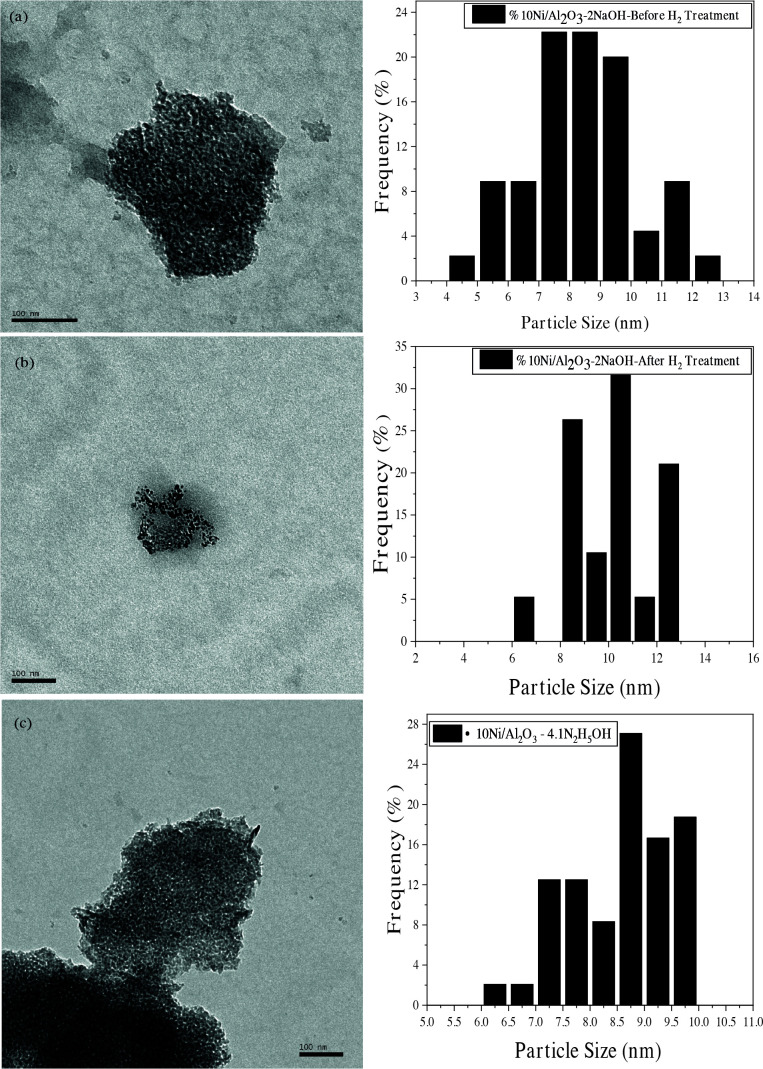
HRTEM views of Ni/γ-Al_2_O_3_-2NaOH catalyst fresh (a) , after H_2_ pre-treatment (b) and Ni/γ-Al_2_O_3_-4.1N_2_H_5_OH (c).

The shape, particle size and morphology of single phase Ni nanoparticles examined by HRTEM analysis are shown in Figure 8. The HRTEM images belong to fresh and after H_2_ pre-treatment of Ni/γ-Al_2_O_3_-2NaOH catalyst and Ni/γ-Al_2_O_3_-4.1N_2_H_5_OH, respectively. It can be concluded from the images that the catalysts have may still have excess amount of PVP surrounding each Ni nanoparticle. Histograms of the size distribution of nanoparticles in each sample were also created from the HRTEM views provided inset in each figure. The narrow size distribution of Ni-containing catalysts suggests the metal species are well dispersed on the support surface and a large amount of them are spherical and similar nanoparticles not agglomerated with each other due to utilizing not only PVP also NaOH and hydrazine use.

Figure 8a and b point out that the boundaries of nickel particles appeared clearly after H_2_ pre-treatment process. The stability and surface properties of nickel-metal nanoparticles supported on alumina depend on thermal pre-treatment under H_2_ atmosphere before CPOM. The average particle size of catalyst synthesized with the molar weight ratio of NaOH to Ni (2:1) (before H_2_ treatment) was distributed in a range of 4-12.80 nm with the average size around 8.5 nm as seen in its histogram. Besides, Ni/γ-Al_2_O_3_-2NaOH catalyst’s (after H_2_ treatment) average particle size was determined as 10 nm while it was distributed in a range of 6-12 nm. Figure 8(c) also shows the view of the catalyst Ni/γ-Al_2_O_3_-4.1N_2_H_5_OH with its histogram. The untreated catalyst shows a uniform Ni particle dispersion with a size smaller than 10 nm in diameter. The average particle size of catalyst synthesized with the molar ratio of N_2_H_5_OH/Ni=4.1 was determined as 8.4 nm in the distribution range of 6-10 nm as seen in its histogram. 

In terms of the morphology of Ni nanoparticles, a similarity was observed especially in Ni/γ-Al_2_O_3_-4.1N_2_H_5_OH catalyst. According to TEM data, the particle size of Ni in catalysts synthesized by the modified polyol method is approximately 6–10 nm, which is slightly different from the XRD data. This may be a result of the particles not being homogeneous in size, shape, composition and crystallinity. In a study, it is emphasized that the results obtained by Scherrer’s law are mainly dependent on the geometry of the particles; they are also sensitive to the crystalline domains on each particle, to the lattice defects, and the differences in scattering due to the amorphous PVP surface layer [54]. However, in both characterization techniques, particle sizes tend to decrease or increase depending on the varying ratios of PVP, NaOH and hydrazine.

### 3.2. Catalytic activity, selectivity and stability tests 

Catalytic activity and selectivity tests of Ni-alumina supported catalysts were carried out in the GC-micro reactor system. Before the catalytic activity tests, H_2_ pre-treatment was applied to the catalysts to remove impurities from the surfaces for 4 h at 450 °C. Boudjahem et al. [55] synthesized nickel nanoparticles supported on silica by hydrazine reduction in aqueous solution for benzene hydrogenation and exhibited that the freshly synthesized catalysts could be inactive when a hydrogen pre-treatment is not applied. According to the literature and our previous studies [41] that is probably attributed to the organic matrix content coming from the acetate fragment of the Ni salt, ethylene glycol, very small amount of NaOH and mainly sourced from PVP remaining on the fresh catalysts surface at the end of the washing process. This is also confirmed and demonstrated by TGA analysis. This organic matrix blocks the access of the active nickel sites. With the hydrogen treatment, the organic matrix and other residuals are removed and facilitate the liberation and accession of the Ni active sites on support, increasing the activity and selectivities of catalysts. 

ours;To investigate the influence of hydrazine and NaOH on catalytic activity, the Ni/γ-Al_2_O_3_-1NaOH, Ni/γ-Al_2_O_3_-2NaOH, Ni/γ-Al_2_O_3_-4NaOH, Ni/γ-Al_2_O_3_-4.1N_2_H_5_OH and Ni/γ-Al_2_O_3_-8.2N_2_H_5_OH catalysts were tested in the catalytic partial oxidation of methane (CPOM) to syngas at temperatures between 600 and 800°C.

**Figure 9:**
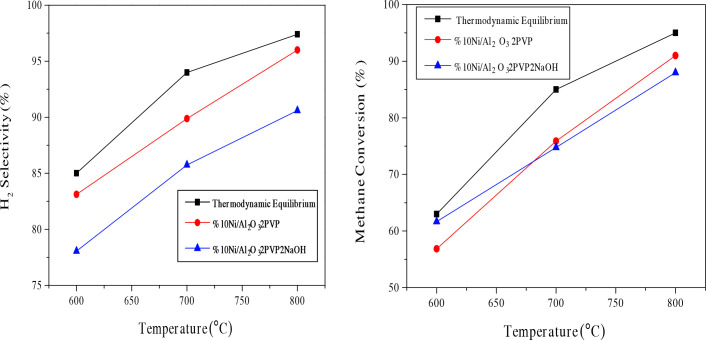
Activity and H_2_ selectivity results of Ni/γ-Al_2_O_3_-2NaOH catalyst between 600 and 800  C. Catalyst: 40 mg. GHSV:157,500 Lkg^-1^ h^-1^ CH_4_:O_2_:N_2_=30:15:60 P:1 atm.

**Figure 10:**
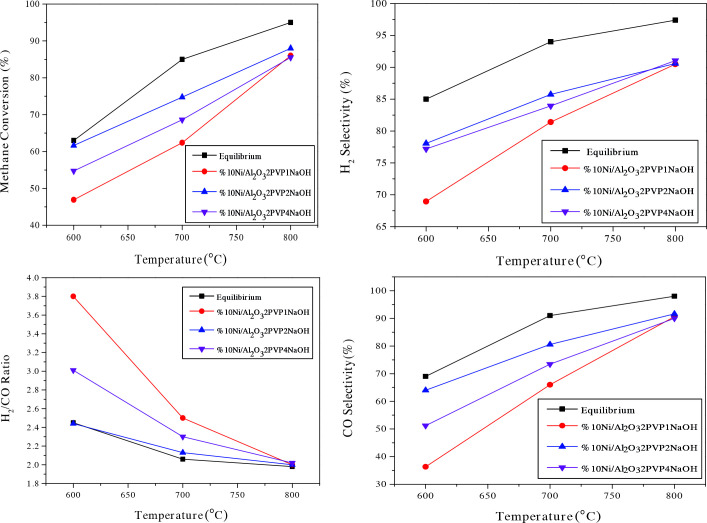
Activity, selectivity and H_2_/CO results of pre-treated Ni/γ-NaOHAl_2_O_3_ catalysts between 600 and 800  C. Catalyst: 40 mg GHSV:157,500 Lkg^-1^ h^-1^ CH_4_:O_2_:N_2_=30:15:60 P:1 atm.

Oxygen was totally used for all temperature ranges; only H_2_ and CO selectivities and CH_4_ conversions are shown in the Figure 9, Figure 10 and Table 3, Table 4. Thermodynamic equilibrium values were provided from Enger et al.[56]. Figure 9 shows methane conversion and H_2_ selectivity results of the pre-treated Ni/γ-Al_2_O_3_-2PVP-2NaOH and Ni/γ-Al_2_O_3_-2PVP catalysts which are comparatively given between 600 and 800°C. Ni/γ-Al_2_O_3_-2PVP catalyst displayed higher conversion, ranging between 75.80 and 91% CH_4_ conversion at 700800 C due to higher Ni loading efficiencies.

**Table 3:** Catalytic activity comparison of Ni/γ-Al2O3-aNaOH catalysts in CPOM.

Catalyst Ni/γ-Al2O3-2PVP	Reaction Temperature (°C)	CH4 Conversion (%XCH4)	H2 Selectivity (%SH2)	CO Selectivity (%SCO)	H2/COratio
1NaOH	700	62.40	81.40	66.00	2.50
	800	86.00	90.50	90.60	2.00
2NaOH	700	74.76	85.75	80.54	2.13
	800	88.00	90.60	91.64	2.00
4NaOH	700	68.63	83.95	73.42	2.30
	800	85.52	91.05	89.97	2.02

Reaction conditions: CH4/O2/N2=2:1:4 (105 mL/min) GHSV (l/kg h) = 157,500 pressure: 1atm

**Table 4:** Catalytic activity comparison of Ni/γ-Al2O3-bN2H5OH catalysts in CPOM.

CatalystNi/γ-Al2O3-2PVP-2NaOH	Reaction Temperature (°C)	CH4 Conversion (%XCH4)	H2 Selectivity (%SH2)	CO Selectivity (%SCO)	H2/COratio
4.1N2H5OH	700	69.90	84.36	75.64	2.23
	800	82.50	89.35	87.91	2.03
8.2N2H5OH	700	58.00	77.23	66.59	2.32
	800	72.50	85.10	82.63	2.06
20.5N2H5OH	700	61.45	70.20	59.89	2.34
	800	76.76	77.93	74.65	2.08

Note: Starting from 500 ° C using reduced catalyst, measurement was taken to 800 °C every 100 °C and only the last two temperatures are given in the table.

Among the samples prepared in a highly basic medium with the addition of NaOH, the Ni/γ-Al-º_2_O_3_-2NaOH catalyst with a crystalline size of 13.50 nm had the highest methane conversion while the Ni/γ-Al,_2_O_3_-4NaOH catalyst, having the similar crystallite size, had the highest hydrogen yield. t the reaction temperature of 800 °C, the maximum methane conversion belongs to Ni / γ-AlWith a value of about 88% a _2_O_3_-2NaOH catalyst. The Ni supported catalyst prepared with a NaOH/Ni molar ratio (4:1) showed a lower methane conversion (85.52%) compared to the Ni/γ-Al_2_O_3_-1NaOH and Ni/γ-Al_2_O_3_-2NaOH catalysts. However, in the catalysts prepared with three different NaOH molar ratios, there was not much difference in terms of methane conversion percentage at 800C .For Ni /γ-Alº_2_O_3_-2NaOH catalyst at 800 °C temperature, 2.00 H_2_/CO ratio, 91.64% CO selectivity and 90.60% H_2_ selectivity were obtained. The reaction results of the catalysts for 700 C and 800 C are given in detail in Table 3 and Table 4.ºº

**Figure 11:**
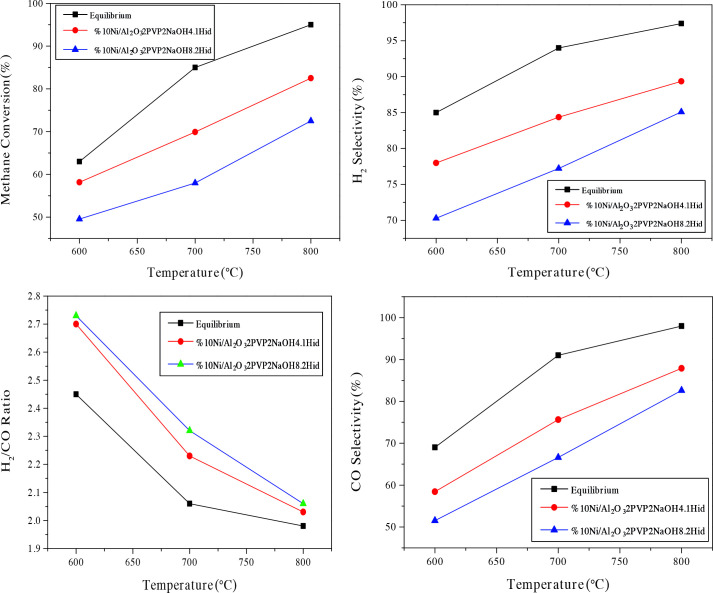
Activity, selectivity and H_2_/CO results of pre-treated Ni/γ-Al_2_O_3_ 4.1N_2_H_5_OH and Ni/γ-Al_2_O_3_-8.2N_2_H_5_OH catalysts between 600 and 800  C. Catalyst: 40 mg GHSV:157,500 Lkg^-1^ h^-1^ CH_4_:O_2_:N_2_=30:15:60 P:1 atm.

Figure 11 belongs to the activity, selectivity, and H_2_/CO results of the pre-treated Ni/γ-Al_2_O_3_-4.1N_2_H_5_OH and Ni/γ-Al_2_O_3_-8.2N_2_H_5_OH catalysts, between 600 and 800°C. 

At temperature 800°C, H_2_/CO ratios of 2.03 and 2.06, 87.91% and 82.63% selectivities of CO and 89.35% and 85.10% selectivities of H_2_ were obtained for Ni/γ-Al_2_O_3_-4.1N_2_H_5_OH and Ni/γ-Al_2_O_3_-8.2N_2_H_5_OH catalysts, respectively. Compared to catalysts prepared without hydrazine, there was no increase in activity and selectivity although the smallest crystallite sized-catalysts were determined on them. This was probably due to the lower amounts of Ni loading onto alumina with the increasing ratio of hydrazine, as mentioned before. Also, nickel loading amounts and hence catalytic activity may be affected due to magnetic interaction. From another point of view, hydrogen chemisorption is one of the most widely used methods, especially to investigate the chemical surface properties of metal supported catalysts. For supported nickel catalysts reduced with gaseous hydrogen prior to reaction, the amount of hydrogen adsorbed is considered to be a measure of the metal dispersion. In addition, as a result of the formation of more than one active center in such metal catalysts, Hgood _2_-TPD profiles with two or more reduction peaks are formed [57]. As we know from previous studies, the supported Ni catalysts, like other transition metal catalysts, exhibit good hydrogen reservoir properties and can adsorb and store large amounts of H_2_ [58]. Boudjahem et al. [59] conducted a study to directly demonstrate the effect of nickel supported catalysts on metal phase morphology on surfaces and catalytic properties [60]. They stated that the thermal stability of the whisker-like nickel phase under hydrogen atmosphere was dependent on the preparation method, which was supported on silica and obtained by reduction of nickel acetate in aqueous hydrazine medium. From the hydrogen adsorption (H_2_-adsorbtion) and desorption (H_2_-TPD) studies, two desorption peaks were found, namely low temperature ( -300C) and high temperature (º-300C), depending on the weak or strong binding of hydrogen to the active nickel site. They found that whisker-like Ni adsorbs and stores more hydrogen than spherical Ni [55]. Since the morphology of Ni nanoparticles synthesized in our study is closer to spherical particles, obtaining up to 86% methane conversions in the partial oxidation reaction of methane may be related to more limited hydrogen storage, that is, limited nickel dispersion. The activity and selectivity results of these catalysts are lower than expected although the catalytic activity and selectivity results could be acceptable in the perspective of thermodynamic equilibrium.

ºAs can be seen from Figure 10, Table 3 and 4, in reaction tests of all catalysts, CH_4_ conversion, H_2_ and CO selectivity values were generally obtained lower than equilibrium values below 800 °C. First of all, Ni metal particles can be oxidized by a significant flow of oxygen over the catalyst surface due to the high space velocity. The reaction rate of the oxidation reaction is higher than the dissociation of CH_4_ at low temperatures (-650°C). The oxidized Ni surface enhanced the total oxidation reaction, causing for a significant decrease in both CH _4_ conversion and selectivity to H_2_ and CO. Secondly, under such a high space velocity and low temperature as 600 or 700°C, endothermic steam and dry reforming reaction rates inhibited the thermodynamic equilibrium and caused less product. This means that the dry reforming reaction rate is relatively more dependent on temperature. According to these results, while methane firstly gives a complete combustion reaction with oxygen, the water and carbon dioxide formed react with the remaining methane to form hydrogen and carbon monoxide at higher temperatures. It is believed that dry reforming reaction rate was low compared to other reforming reaction at low temperatures, thus causing less CO formation. Therefore, H_2_/CO ratios could be more than the equilibrium value for the kinetically inhibited conditions at low temperatures [41]. As can be understood from the Figure 10, given H_2_/CO ratios were much higher than equilibrium values at 600 and 700°C and these support this assertion.

Among the prepared catalysts, stability test was performed for methane partial oxidation reaction at 800 °C to Ni /γ-Als _2_O_3_-4.1N_2_H_5_OH catalyst with low Ni crystallite size 10 h. This catalyst was preferred because it can be prepared at much lower temperatures with the modified polyol method and gives high methane conversion. Although high temperature and excess methane reactant mixture was used for the catalyst, no tragic deactivation (activity loss of about 1%) was observed during the 10h reaction in the catalyst.

**Figure 12:**
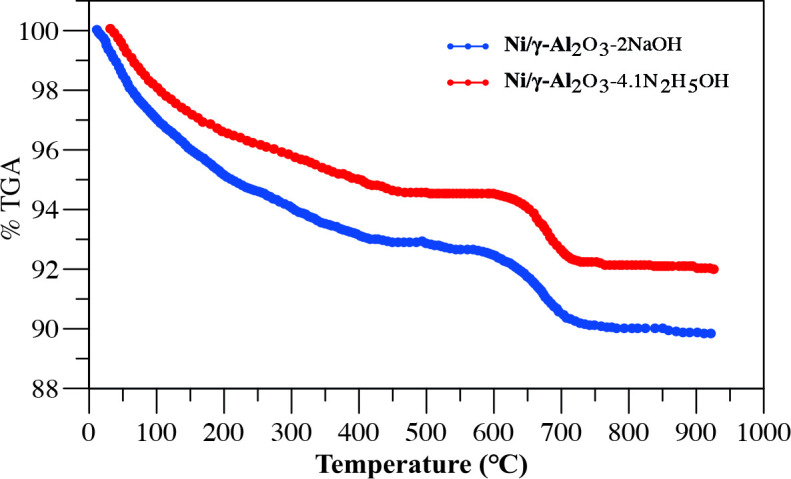
TGA analysis of Ni/γ-Al_2_O_3_-4.1N_2_H_5_OH Ni/γ- Al_2_O_3_-2NaOH after CPOM reaction at 800  C.

The formation of carbon during the CPOM reaction was investigated by thermogravimetry analyses of the spent catalyst after the 10h stability test at 800during ours our,C. Figure 12 shows the thermograms obtained in the oxidation of the carbon deposits formed during CPOM on Ni/γ-Alº_2_O_3_-4.1N_2_H_5_OH and Ni/γ-Al_2_O_3_-2NaOH catalysts. The TGA profile for the spent catalyst ,evidences a total weight loss of 9.45%, much higher than for the Ni/γ-Al_2_O_3_-4.1N_2_H_5_OH catalyst. Moreover, two different weight loss stages can be observed for Ni/γ-Al_2_O_3_-4.1N_2_H_5_OH catalyst; a first step corresponding to around 5% of weight loss due to moisture and the other organic residues, and a second step corresponding to 2.25% weight loss at temperature between 500and 700C, ascribed to the oxidation of to amorphous carbon deposited on the catalyst.

A small hump was observed during the weight loss of the catalyst Ni/γ-AlºC º_2_O_3_-4.1N_2_H_5_OH in the temperature range 600-650 °C, which can be attributed to the oxidation of metallic Ni particles. It should be noted that in the literature [61], in the Ni/CeO_2_(HT) catalyst prepared by hydrothermal synthesis, the weight loss corresponding to the accumulated carbon is approximately 15% and still stable during the CPOM reaction. Also perovskites with formula LaNiby weight _1-x_Co_x_O_3_ (x = 0.0, 0.2, 0.5 and 1.0) were investigated in the CPOM reaction [62]. It should be noted that the methane conversion of all prepared catalysts varies between 6074%, which is below the conversion and selectivity values we obtained in our study, and graphitic carbon was detected in all samples. According to our results, which are shown in Figure 13, no serious activity loss occurred in catalyst Ni/γ-Al-_2_O_3_-4.1N_2_H_5_OH under accelerated deactivation conditions in accordance with the literature.

## 4. Conclusion

The influence of the preparation method for the synthesis of of PVP-stabilized Ni/Al_2_O_3 _nanoparticles for methane partial oxidation (CPOM) was evaluated in terms of catalytic activity, selectivity and stability.The preparation of nickel nanoparticles was carried out by the reduction of nickel acetate with aqueous hydrazine at a temperature of 80 C and a pH of 10º12 and only in the presence of a NaOH at ap192C.

-x.ºThe powder XRD results showed that the peaks correspond to metallic nickel particles with fcc structure. HRTEM images show that the non-agglomerated Ni nanoparticles are well-dispersed on alumina support, and the average particle size was determined in the range of 8.48.5 nm. Among the Ni/Al-_2_O_3_ catalysts prepared by alkaline polyol and hydrazine reduction method, the catalysts, with a molar ratio of NaOH/Ni:2 and N_2_H_5_OH/Ni=4.1 showed methane conversions about 88% and 82% at 800 °C. 

Finally, nickel catalysts in the nanometer size range with a fine size distribution was synthesized by the modified polyol process, which combines some advantages of polyol and chemical reduction method. The method allows better control of the final materials’ morphology, their physical and chemical properties. The catalytic activitiy and stability of Ni catalysts synthesized by the modified polyol method for CPOM reaction are within acceptable limits, and the cost and reaction time have been reduced. No signal of deactivation by coke formation, such as confirmed by XRD and TG analysis carried out after the catalytic tests. Still, further work is required .
